# The risk matrix approach: a helpful tool weighing probability and impact when deciding on preventive and diagnostic interventions

**DOI:** 10.1186/s12913-022-07484-7

**Published:** 2022-02-17

**Authors:** Stéphanie M. P. Lemmens, Veronica A. Lopes van Balen, Yvonne C. M. Röselaers, Hubertina C. J. Scheepers, Marc E. A. Spaanderman

**Affiliations:** 1 Department of Obstetrics and Gynecology, School for Oncology and Developmental Biology (GROW), Maastricht University, Universiteitssingel 50, Maastricht, 6229 ER The Netherlands; 2 Department of Obstetrics and Gynecology, Maastricht University Medical Center, Maastricht, The Netherlands; 3 Limburg Obstetric Consortium, ROS Robuust, Eindhoven, The Netherlands

**Keywords:** Risk matrix approach, Decision-making, Probability, Impact

## Abstract

**Background:**

Clinical guidelines are developed to lower risks, mostly viewed upon as probability. However, in daily practice, risk is perceived as the *combination* of probability and the impact of desired and adverse events. This combination of probability and impact can be visualized in a risk matrix. We evaluated the effect of interventions and diagnostic thresholds on modeled risk, by using the risk matrix approach (RMA) in a clinical guideline development process, and investigated which additional factors affected choices.

**Methods:**

To improve care outcomes, we developed new guidelines in which care professionals had to decide upon novel interventions and diagnostic thresholds. A risk matrix showed the probability and impact of an intervention, together with the corresponding risk category. First, professionals’ opinion on required performance characteristics on risk were evaluated by a qualitative online survey. Second, qualitative assessment of possible additional factors affecting final decisions, that followed from group discussion and guideline development were evaluated.

**Results:**

Upfront, professionals opinioned that non-invasive interventions should decrease the general population risk, whereas invasive interventions should decrease the risk in high-risk groups. Nonetheless, when making guidelines, interventions were introduced without reaching the predefined threshold of desired risk reduction. Professionals weighed other aspects besides risk reduction, as financial aspects and practical consequences for daily practice in this guideline-making process.

**Conclusion:**

Professionals are willing to change policies at much lower level of anticipated effectiveness than defined upfront. Although objectively presented data structured group discussions, decisions in guideline development are affected by several other factors than risk reduction alone.

## Background

During clinical guideline development, healthcare professionals discuss risk and usually weigh the probability of an event occurring. However, *risk* is generally described as the *combination* of the linked likelihood of occurrence times the impact of a certain event [1, 2]. The impact of that event can be either beneficial or detrimental, ranging from minor to major. Therefore, decisions are affected by estimating chances and taking the characteristics of these taken chances into account. Nonetheless, risk is an objective existence which can never be eliminated, is abrupt and often harmful with people suffering loss, may be uncertain in whether and when the event will happen, and is constantly developing with science and technology [3].

In healthcare, prior to introducing a new intervention or diagnostic threshold, professionals and policy makers have to consider what level of risk they would accept and what the expected effect of a newly introduced intervention is. Some interventions have a diagnostic character, whereas others have a preventive or therapeutic effect. A newly introduced intervention may decrease the probability of an event to happen and/or can have an effect on its impact. In any case, an intervention is expected to change risk. When deciding upon new interventions or strategies, these novel actions should at least prevent or lower the risk of an adverse event to occur. Nonetheless, given the paradigm, it may be that professionals, policymakers or even patients may decide differently on the same questions raised.

A risk matrix can be helpful and insightful to agree upon new or changed interventions as they present complex risk data in a concise visual and mathematical way. Using a risk matrix is both a qualitative and quantitative approach to prioritize risk and start interventions to mitigate the risk and to facilitate constructive discussions within a decision process [1, 4]. The aim of any risk evaluation tool is to ensure that the decision process is transparent, based on best knowledge and reflecting the common understanding of stakeholders [1]. We evaluated the effect of interventions and diagnostic thresholds on risk, using a risk matrix approach (RMA), and factors influencing the decision-making process towards the development of new guidelines and protocols.

## Methods

### Participants

A group of obstetric professionals (*n* = 131) participated from 2013 to 2016 in a guideline development project in the catchment area of the tertiary Maastricht University Medical Center, the Netherlands [5].

### Decision-making process

To make decisions and reach consensus on (new) interventions and diagnostic thresholds, we used the novel ACCORD-tool; Agreement Conform Current Operational Rules and Directives-tool and the risk matrix approach [5]. The ACCORD-tool consists of a four-step bottom-up approach: first summarize current evidence on a specific topic based on existing guidelines, second translate evidence into statements, third send statements in an online survey (Survey Monkey, Palo, Alto, CA, USA) to the professionals to rank statements by level of agreement on a 10-point Likert-scale (1 totally disagree - 10 totally agree), and fourth review the survey results within a team of mandated representatives to make decisions on best given care. Survey results were expressed in statement means with standard deviations (SD). In these, statements with mean between 3 and 8, or SD > 2 were discussed, a mean below 3 or above 8 and a SD < 2 could be immediately rejected or accepted, respectively. Decisions were agreed upon when at least 85% of the mandated team was in favour.

**Fig. 1:**
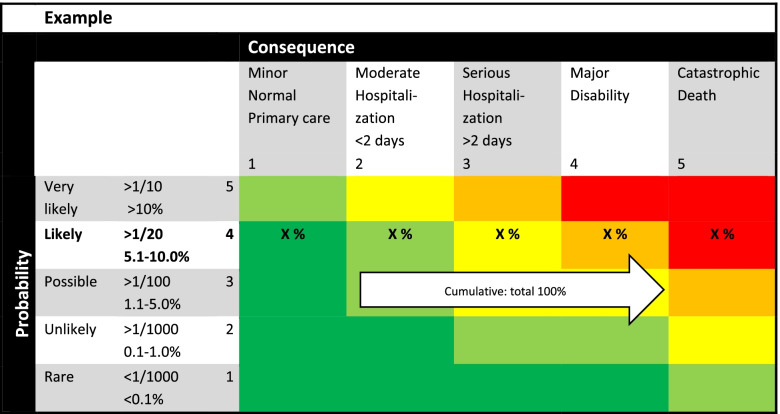
Example of a risk matrix

For specific topics, we used the RMA. The design of a risk matrix is a basic and often used concept to evaluate risks, which was adapted to our situation and interventions [1]. Our risk matrix consists of five rows and columns, which define categories of probability (likelihood) and impact (consequences). The rows of the risk matrix (Fig. 1), numbered 1 to 5, represent probability ranging from rare (< 0.1%) to very likely (> 10%) [4]. The columns, numbered 1 to 5, represent impact ranging from minor to catastrophic, resulting in death. We added healthcare related labels to describe impact, but these can be adjusted depending on the profession.

**Fig. 2:**
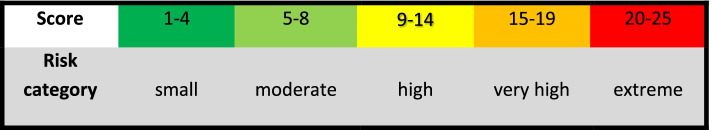
Risk categories with corresponding scores and colors

Multiplication of the ordinal numbers of the probability and impact category results in a riskscore, presented as risk categories with corresponding colors (Fig. 2). A higher score indicates a higher risk category. We considered risk-score 1-4 a small risk (dark green), risk-score 5-8 a moderate risk (light green), risk-score 9-14 a high risk (yellow), risk-score 15-19 a very high risk (orange) and risk-score 20-25 an extreme risk (red). To assess the change in risk of any intervention, the cells within the matrix contain percentages, estimates based on existing data and knowledge, given the reported chance [6, 7]. The cumulative percentage of all cells in one row adds up to 100%. For each intervention, the median population risk (p50) and corresponding p5 and p95 were assessed.

### Interventions

Using the online survey, we first investigated the expectations of professionals on risk reduction regarding non-invasive, moderate-invasive and very-invasive interventions. Second, to explore if the RMA affected the decisions taken in guideline development, we evaluated the effect on risk reduction with a preventive and diagnostic intervention. As a preventive intervention we chose to evaluate the risk of preeclampsia with and without calcium supplementation throughout gestation [7, 8]. The World Health Organization and Dutch Health Council recommend pregnant women with low calcium intake to take 1 g calcium supplementation a day [9, 10]. Calcium supplementation is not part of standard antenatal care in The Netherlands, despite the fact that calcium is considered a non-invasive intervention reducing the probability of developing preeclampsia significantly [7, 11]. A Dutch cohort study shows that approximately one third of pregnant women has a dietary calcium intake below 1 g/day and, as such, at potential benefit of extra calcium [8, 12].

The clinical effect changing the ultrasonographically determined fetal weight *threshold* that defines fetuses small for gestational age (SGA) from the 10th to the 5th growth-chart centile (p). Changing the threshold was seen a diagnostic intervention. SGA is associated with an increased risk of perinatal morbidity and mortality, especially when not detected prior to delivery [6, 13]. The most commonly used definition to describe SGA is an estimated fetal weight or abdominal circumference measured by ultrasound below the 10th centile [6, 14]. To detect those at highest risk in order to change the intensity of given care, we redefined and reorganized the diagnostic process and follow-up of SGA fetuses.

For both interventions, professionals had to decide on several questions. First, does the intervention need to decrease the probability of occurrence (chance), or result in a less severe impact (consequence) and/or a decrease in risk category? Second, how much change in chance, consequence or risk category is desired or required to value a new intervention valid? Third, should the new intervention be effective for the whole population or only to individuals at highest risk of adverse outcome? Fourth, are there other factors affecting individual’s professional decisions when deciding upon new-to-introduce interventions?

## Results

**Fig. 3:**
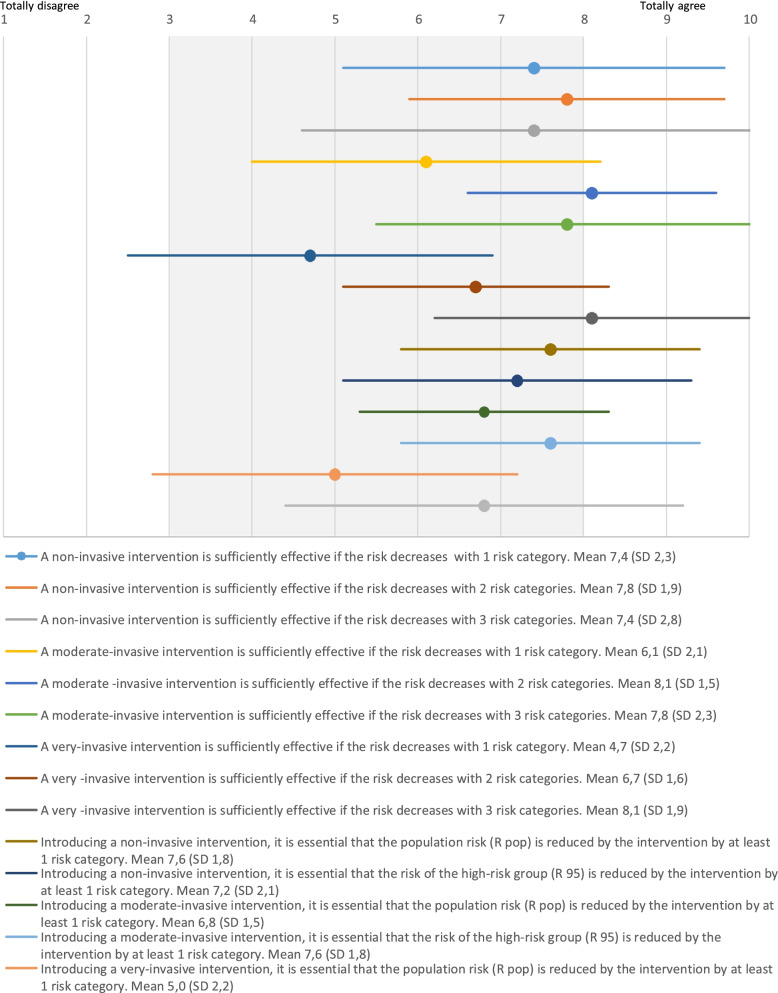
Statements with mean and SD for risk reduction

Upfront, participants opinioned that non-invasive interventions and moderate-invasive interventions should decrease the risk of an event with two risk categories (mean 7.8, SD 1.9 and mean 8.1, SD 1.5), and for very-invasive interventions even three risk categories (mean 8.1, SD 1.9) (Fig. 3). Non-invasive interventions should decrease the population risk (mean 7.6, SD 1.8), whereas moderate and very invasive interventions should decrease the risk of the highest-risk group (mean 7.6, SD 1.8 and mean 6.8, SD 2.4). Even though these expectations on risk reduction were not met, calcium supplementation and the 10th centile as threshold for diagnosing SGA were still introduced in the new developed guidelines. In this decision-making process participants weighed other aspects besides risk reduction, such as financial aspects, invasiveness of the intervention and consequences for daily practice.

### Considerations regarding calcium supplementation as preventive intervention for preeclampsia

#### Survey

**Fig. 4:**
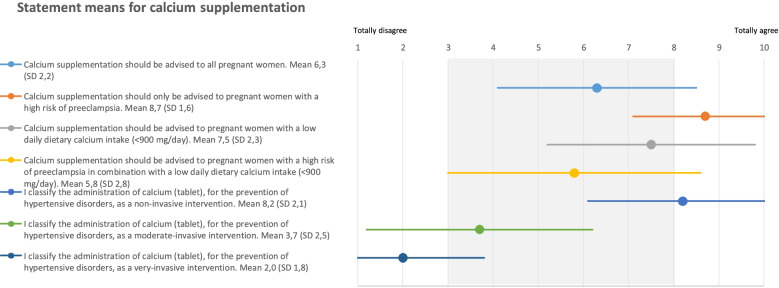
Statements with mean and SD for calcium supplementation

Before discussing the new strategy, only 38% of the consulted gynecologists and midwives (*n* = 50) responding to the survey, advised extra calcium supplements to their pregnant patients. Of those professionals who advised women to take calcium supplementation, 70% advised calcium only to women with a high risk of preeclampsia and 30% only to women with a low dietary calcium intake or in combination with a high risk of preeclampsia. Figure 4 presents the statements regarding calcium supplementation with the corresponding mean and SD. The statement regarding calcium supplementation only for pregnant women with a high risk of preeclampsia (mean 8.7, SD 1.6) were most agreed upon. The professionals classified calcium as a non-invasive intervention (mean 8.2, SD 2.1).

#### Risk matrix

**Fig. 5:**
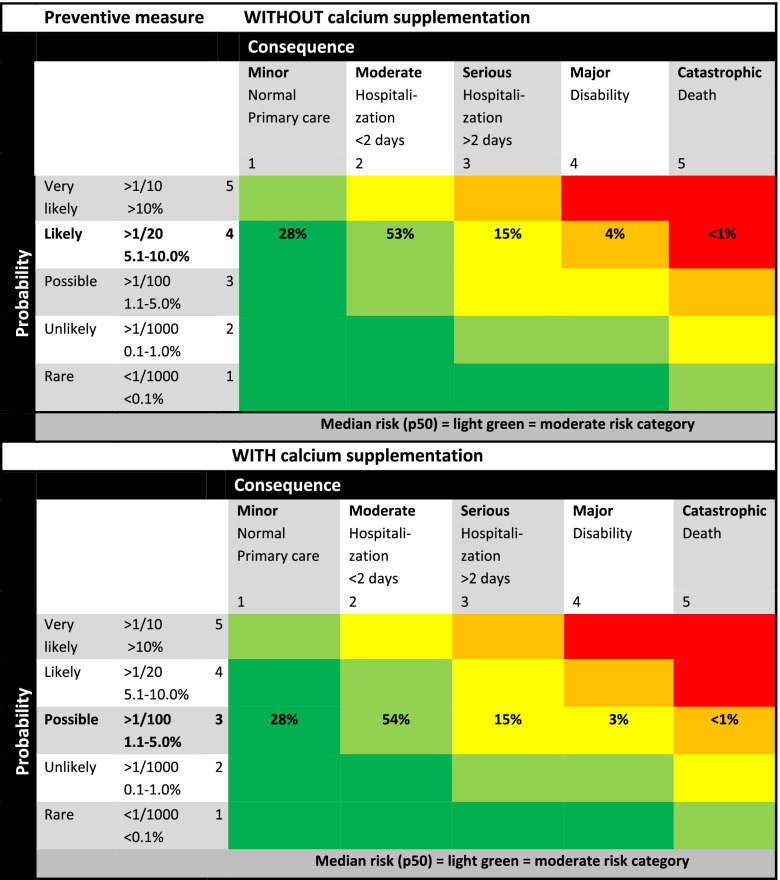
Risk matrix approach displaying the risk of preeclampsia and maternal consequences for the situation without and with calcium supplementation

Figure 5 shows probability, impact and associated risk categories for developing preeclampsia in pregnant women without and with calcium supplementation [6]. In women *without* calcium supplementation the probability of developing preeclampsia is 6.5%, classified as ‘likely’ (5.1-10%). The corresponding percentage of maternal death in this group is 0.1 and 4.3% suffers from serious maternal morbidity (composite outcome eclampsia, renal failure, HELLP syndrome and admission to intensive care). Weighing the effects of hypertension and preeclampsia, gestational diabetes, instrumental delivery, infection and gestational age at birth and birthweight from our national birth control registry, we estimated that of all women, 28% would deliver under supervision of their midwife in primary care, the vast majority, 53%, would be hospitalized for less than 2 days, and 15% will be hospitalized for more than 2 days. For women who are not taking calcium supplements, the overall median (p50) risk of preeclampsia and corresponding P5-P95 is 8 (4-12). This risk is classified as moderate risk (Fig. 2) and calculated by multiplying the probability category 4 and the consequence category 2 (Fig. 5).

If all pregnant women take supplemental calcium, preeclampsia will occur in 4.8% of women, reducing the probability of preeclampsia from ‘likely’ to ‘possible’ (1.1-5%). Maternal death reduces to 0.02% and severe maternal morbidity to 3.4% of cases. Of all women, we estimate that 28% will deliver under supervision of their midwife in primary care, the vast majority of 54% will be hospitalized for less than 2 days and 15% will be hospitalized for more than 2 days. Due to the decrease in probability, the risk category changes for ‘disability’ from very large (orange) to large (yellow) and for ‘death’ from extreme (red) to very large (orange). For women using calcium supplements, the overall median (p50) risk of preeclampsia and corresponding P5-P95 is less than without calcium supplementation but still 6 (3-9), also classified as a moderate risk (Fig. 2).

#### Argumentation

Although the intervention did change the population’s risk as it did not alter risk category, nonetheless, professionals decided to introduce calcium supplementation as new intervention to all pregnant women. This decision was made, because calcium supplementation during pregnancy was seen as non-invasive and safe intervention, well tolerated by pregnant women. Moreover, it was argued that it lowered the incidence of preeclampsia especially in women with a low dietary calcium intake (< 1000 mg/day), which accounts for approximately 30% of the Dutch population [8]. Moreover, the *absolute* chance on the worst outcome, disability or death, became smaller. Besides being beneficial in reducing the incidence of preeclampsia, it also may have a positive effect on related health care costs [8].

### Considerations regarding different fetal growth thresholds to diagnose SGA as diagnostic intervention

#### Survey

**Fig. 6:**
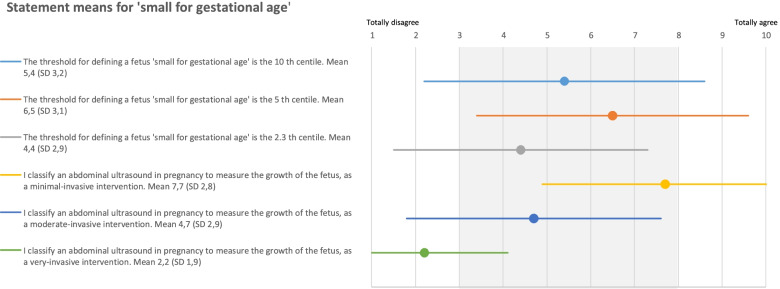
Statements with mean and SD for the diagnostic SGA threshold

Figure 6 presents the statement scores for the 10th, 5th and 2.3th fetal growth centile, which show a slight preference for the 5th centile as cut-off value (mean 6.5, SD 3.1). Performing an ultrasound to detect an SGA fetus, is classified by professionals as a non-invasive intervention (mean 7.7, SD 2.8). Professionals opinioned that newly introduced non-invasive interventions should decrease the risk of an event with two risk categories (mean 7.8, SD 1.9) and to decrease the population risk with at least one risk category (mean 7.6, SD 1.8).

#### Risk matrix

**Fig. 7:**
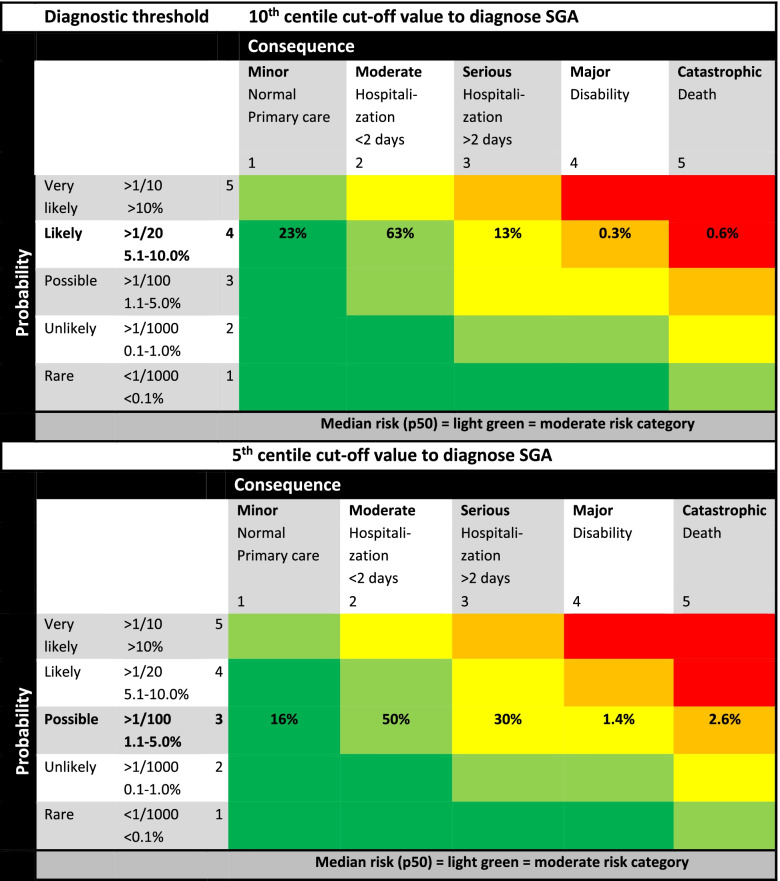
Risk matrix approach displaying the differences in neonatal consequences for small for gestational age fetuses, using the 10th and 5th centile as threshold for the fetal abdominal circumference measured by ultrasound [15]

Figure 7 presents the probability, impact and associated risk categories for the 10th and 5th centile [15]. After consultation with the professionals, we left the 2.3th centile out of consideration for the risk matrix approach as the 10th and 5th centile were focus of discussion. Of all fetuses, 10% has an estimated weight by ultrasound below the 10th centile, classified as SGA. After delivery, based on our national birth registry, 23% of these neonates will be taken care of in primary care, 63% of these neonates will be admitted to the hospital for less than 2 days, 13% for 2 days or more, 0.3% will have some degree of disability and the mortality rate is approximately 0.6%.

For all fetuses with an estimated growth below the 10th centile, the overall median (p50) risk of complications and corresponding P5-P95 is 8 (4-12), classified as moderate risk with 0.9% chance on major disability and death (Figs. 2 and 7). Choosing the 5th centile as cut-off value for fetal growth restriction, the probability decreases from category ‘likely’ (5.1-10%) to ‘possible’ (1.1-5.0%). Of these neonates 16% will be taken care of in primary care, 50% will be hospitalized less than 2 days, 30% for 2 days or more, 1.4% will have some degree of disability and the mortality rate is around 2.6% [7]. For all fetuses with a growth below the 5th centile, the overall median (p50) risk of complications and corresponding P5-P95 is 6 (3-9), classified as moderate risk (Figs. 2 and 7), but at the expense of a 4% risk on major disability and death. Decreasing the cut-off value from the 10th to the 5th centile therefore results in a smaller population risk to be diagnosed SGA but a higher chance on severe consequences.

#### Argumentation

Despite the survey results indicate a preference for the cut-off value for detection of SGA at the fifth centile, the professionals chose the 10th centile after group discussion. They decided in favor of a more sensitive threshold, to detect possible growth restricted fetuses at an early stage in order to prevent severe neonatal consequences. Especially, because of the Dutch situation, in which labor of an undetected SGA fetus without continuously fetal monitoring in a home birthing setting may relate to more offspring morbidity and death [13]. Despite the fact that performing the ultrasound is classified as a non-invasive intervention, the uncertainty for the future parents that comes with the suspicion SGA is seen as an unwanted side effect. Below this centile, healthcare professionals have to take measures for follow-up (new appointments, repeating ultrasound examination etc.). This implicates more intensified surveillance and consequently, for the Dutch situation, shifting care from midwife-led to gynecologist-led. This transfer of care from midwife to gynecologist automatically results in financial consequences on population level, but also on an individual professional level.

## Discussion

Although by most clinicians, risk is viewed upon as chance, in decisional processes, risk describes the combination of probability and impact of an event. In designing and constructing guidelines, risk matrices could be pivotal to present data objectively, to structure group interactions and to come to balanced choices [16]. Before deciding upon new interventions, we first investigated the professional’s opinion on risk reduction by newly introduced interventions and thresholds. The participants opinioned that newly introduced interventions and thresholds should decrease the risk of an event with two to three risk categories. Furthermore, non-invasive interventions should reduce risks on population level, not only in the highest risk group. Nonetheless, despite these expectations on risk reduction, calcium supplementation and a higher cut-off value for diagnosing SGA were introduced. This indicates that decisions made to delineate guidelines are affected by changes in multi-dimensional risk, but apparently also depend on several other factors, in which lowering the most extreme unwanted but preventable outcome, financial aspects, practical consequences for daily practice were most dominant.

Besides the actual risk, risk perception also influences decision-making. Risk perception refers to the people’s subjective assessment of the probability of a specified type of accident happening and its possible impact [17–19]. The subjective judgement of risk includes personal beliefs, attitudes, experiences and feelings, irrespective of their validity, but also involves people’s social and cultural background [17, 19, 20]. Perception of risk does not discriminate between risk knowledge on the one hand and the value judgement about its acceptability or tolerability on the other hand [20]. People usually underestimate continuous everyday risks as they are less salient compared with dread risks that may be overestimated, even if both cause the same number of fatalities [17]. Several factors can bias risk perception, such as memorability, age, or media coverage of an event [17]. Overall, important in assessing risks is the ease with which people can imagine how risk materializes in daily practice [21]. Experience with specific risks and memories in specific situations contributes to a more realistic risk perception [17].

We presented both interventions in two separate risk matrices, to show the effect of each intervention to experts that are constructing professional guidelines. The risk matrix without the intervention is seen as reference point. The effect of calcium on the prevention of preeclampsia is less evident than the effect of changing the threshold for SGA. Nevertheless, it took less time for the professionals to decide upon calcium supplementation to be implemented into the guidelines, rather than reaching consensus on the threshold for diagnosing SGA. During the decision process, it became clear that for each intervention different aspects were relevant. On the one hand, despite the absent change in population risk category with and without calcium supplementation, extra calcium given to all pregnant women is expected to cause a substantial reduction on population level in the incidence of preeclampsia as well as related health care costs [8]. Moreover, calcium supplementation is viewed upon as relatively inexpensive intervention, side-effects are uncommon and if any usually mild, and the professionals in our project classified calcium as a non-invasive intervention [8]. On the other hand, by changing the threshold for SGA from the 10th to the 5th centile, the population risk lowered, but at the expense of more offspring complications. Despite a lower population risk, the professionals decided to set the threshold for fetal growth restriction at the 10th centile. This indicates that other factors, in addition to the populations risk level, were important in this decision-making process. On the one hand, decreasing the diagnostic threshold results in more severe neonatal consequences that could have been prevented. On the other and, setting the threshold at the 10th centile results in intensified surveillance shifting care from midwife-led to gynecologist-led. In deciding what to recommend in the guidelines, numerous factors different from population risk and its extremes were mentioned. First, although an ultrasound was viewed upon as minimal invasive procedure, the risk for parent’s uncertainty that comes with the suspicion SGA and the subsequent medicalization was seen as an undesired side effect. Second, as the professionals decided to diagnose SGA definitively after two subsequent ultrasounds that are below the chosen threshold with a 2 week interval, professionals perceived this period of uncertainty quiet long for the future parents [14]. Third, although not easily mentioned by professionals, in the Dutch reimbursement system, referring more pregnant women from midwife-led to obstetrician-led care would have negative impact on midwifes income and consequently a positive impact on hospitals finances. Weighing the magnitude of the affected population (5%), its financial consequences and the professionals perceived preventable impact on the worst outcome, professionals decided in favor of a more sensitive diagnostic threshold. Especially given the possible effect of undetected SGA on the chosen level of fetal surveillance during labor in the Dutch home birthing situation was considered a very undesired condition.

The Dutch obstetric system is unique with community midwives providing care for women with low risk pregnancies in primary care and clinical trained midwives, residents and gynecologists providing care for women with high-risk pregnancies in secondary and tertiary care [22]. Transfer of care from midwife to gynecologist, is a result of having one or more risk factors for pregnancy-related complications, unexpected abnormal findings and/or the occurrence of complications during pregnancy or childbirth. As a consequence, midwives view pregnancy and delivery as healthy, physiological events whereas gynecologists are more prone to be concerned about adverse events. These different views became evident during our team meetings, when professionals discussed the need and value of medical interventions.

For decisions made with help of the risk matrix, at population level, actual risk rather than risk category was directive, and an increase in extreme risk was also considered an important issue to be taken into consideration, especially as a timely instituted intervention is capable in reducing the risk. Besides risk, hierarchical structures influenced our decision-making process. Midwives experience a power imbalance in their relationships with their specialist colleagues, which can be explained from a historical perspective [23]. Midwives started as autonomous professionals, responsible for all pregnant women, only consulting another caregiver if the child had died during delivery. As a consequence, midwives were afraid of losing their autonomy by introducing new interventions and changes in current thresholds. In addition, the felt magnitude of the intervention for the individual pregnant women and their healthcare professional were valued in deciding.

We used the risk matrices in combination with the ACCORD tool, a bottom-up approach for developing collaborative interprofessional protocols. The ACCORD tool shows many similarities with the ‘Grading of Recommendations Assessment, Development, and Evaluation’ (GRADE) model and the Evidence to Decision Framework (EtDF) which are frequently used when creating a guideline. However, the main goal of the ACCORD tool is to reach consensus on content of care for clinical practice, thereby weighing current professional guidelines and protocols against the opinions and concerns of the participating disciplines [5]. Similar to the GRADE and EtDF, we identified and prioritized topics to be discussed and summarized current evidence on these topics based on existing guidelines. As guidelines represent topics on which professionals, top-down, have already agreed upon, no systematic search in medical databases was performed, as is the case with the GRADE or EtDF [24].

Obviously, the RMA approach comes with limitations, as the decisions taken are explainable but not always directly logically following the matrix. First, the design of the matrix, the number of rows and columns, and the category scaling and labels are commonly used but arbitrary. Moreover, the percentages filled in the matrix are estimates based on existing data, sensitive to changes in time and place. Therefore, there remains a certain level of subjectivity in the presented figures leaving room for individuals interpretation [2]. On the one hand, lack of exactness is unwanted, on the other hand, understanding the different aspects that are weighed during decision making helps to make balanced choices that can be explained to peers and followers. Second, although better than chance alone, a risk matrix still oversimplifies the complexity of risk. Therefore, risk matrices can only be used as a tool to support risk informed decisions, not for making or computing decisions. Assessment of likelihood, consequences and resulting risk scores actually requires subjective interpretation, whereby different users may obtain different ratings of the same quantitative risks [4]. Therefore, we have to accept that subjective decision-making will always be a part of a risk assessment process, independent of the tool used [2]. Third, risk matrices lack any time valuation, whereas the risk of a certain problem to occur in a week might be very different from the risk of the same problem in a year. A risk can be static over time while others can change overnight. For decision-making, time has to be considered as a separate factor additional to the risk matrix. Our time frame is limited to pregnancy. Time is not a concern for our studied interventions, if calcium supplementation is used in an adequate dosage of 1 g/day from 20 weeks of gestation until the end of pregnancy [25]. The risk of preeclampsia is larger in second and third trimester of pregnancy, but this is the case for every pregnant women, independent of calcium use [26]. The threshold for SGA is not time related. However, gestational age is an important factor, affecting neonatal morbidity and mortality rates, when fetal growth-restriction results in induction of labor [27]. Fourth, because it is a two-dimensional interpretation of risk I it cannot account for interventions that in themselves add a risk.

Although a risk matrix tries to overcome the uni-directional perspective of risks only to be quantified as chance by adding the impact of an adverse outcome to the decision making process, poor resolution, errors in category assignment, vague input and output may still affect resource allocation [28]. There are alternatives to the RMA that make fewer assumptions but are still not completely able to breakdown the probability and outcome as easily. These include the drug fact box and comparative effectiveness tables [29, 30]. They have advantages, and disadvantages, relative to the RMA. As we used the risk matrix in weighing and discussing possible effects of novel changes in new guidelines regarding anticipated costs and clinical effects, we decided, in an attempt to improve resolution, taking a 5 by 5 matrix instead of a less detailed matrix.

## Conclusion

In guideline development, a risk matrix approach is useful to weigh both probability and impact of an event in a concise way, to detail the effect of a newly introduced intervention on modelled risk, and to stimulate risk informed decisions. Nonetheless, decisions taken not only rely on reducing risk, but also on reducing the most unwanted but hardly prevalent outcome, financial aspects, perceived magnitude of the intervention and practical consequences for daily professional’s practice.

## Data Availability

The datasets used and/or analysed during the current study are available from the corresponding author on reasonable request.

## Abbreviations

RMA: Risk matrix approach
n: Number
ACCORD: Agreement Conform Current Operational Rules and Directives
SD: Standard deviation
p: Centile
SGA: Small for gestational age
HELLP: Hemolysis Elevated Liver enzymes Low Platelet count
